# A new, fast and semi-automated size determination method (SASDM) for studying multicellular tumor spheroids

**DOI:** 10.1186/1475-2867-5-32

**Published:** 2005-11-14

**Authors:** Azita Monazzam, Pasha Razifar, Örjan Lindhe, Raymond Josephsson, Bengt Långström, Mats Bergström

**Affiliations:** 1 Institute of Oncology, Radiology and Clinical Immunology, Uppsala University; 2 Uppsala University, Centre for Image Analysis, Lägerhyddsvägen 3, SE-752 37 Uppsala, Sweden; 3 Uppsala Imanet AB (PET Centre), Uppsala University Hospital, SE-751 85 Uppsala, Sweden; 4 Department of Pharmaceutical Biosciences, Uppsala Biomedical Centre, SE-751 24 Uppsala, Sweden; 5 Department of medical Science, Uppsala University Hospital, SE-751 58 Uppsala, Sweden

## Abstract

**Background:**

Considering the width and importance of using Multicellular Tumor Spheroids (MTS) in oncology research, size determination of MTSs by an accurate and fast method is essential. In the present study an effective, fast and semi-automated method, SASDM, was developed to determinate the size of MTSs. The method was applied and tested in MTSs of three different cell-lines. Frozen section autoradiography and Hemotoxylin Eosin (H&E) staining was used for further confirmation.

**Results:**

SASDM was shown to be effective, user-friendly, and time efficient, and to be more precise than the traditional methods and it was applicable for MTSs of different cell-lines. Furthermore, the results of image analysis showed high correspondence to the results of autoradiography and staining.

**Conclusion:**

The combination of assessment of metabolic condition and image analysis in MTSs provides a good model to evaluate the effect of various anti-cancer treatments.

## Introduction

The growth of tumor cells such as Multicellular Tumor Cultures (MTSs) has led to important insights in tumor biology [1]. MTSs represent an intermediary level between monolayer growing cells and solid tumors in animals and humans [2]. The cytology and morphology of MTSs are close to experimental tumors in mice and tumors in humans, before neovascularisation occurs [3]. In fact, MTSs represent quite realistically the three-dimensional growth and organization of solid tumors and therefore simulate the cell-cell interactions and micro environmental conditions found in the tumors [4,5]. For example, multicellular aggregates develop a central necrosis, similar to that seen in many tumors in vivo.

Size determination and evaluation of growth pattern are two essential aspects when studying the characteristics and behavior of the MTSs. These are important factors in studies in which e.g. the total uptake of radiotracers is evaluated and in therapeutically oriented investigations, where a drug may induce morphological changes in the tumors.

The most commonly used technique to determine size of MTSs is to measure two diameters of the spheroid, using a calibrated ocular micrometer on an inverted microscope. These values are then used to determine the volume approximately. This measurement is both time consuming and imprecise, especially for irregular MTSs. Moreover, MTSs tend to develop a central necrosis, i.e. the volume of viable cells differs from the total volume of the aggregate. It is therefore necessary to have the possibility to evaluate the volume of viable cells as one entity describing the aggregate and the fraction of the total volume constituted by viable cells as another entity.

In the present study an effective, fast and semi-automated method (SASDM) for more accurate determination of the size of MTSs and its fraction of viable cells was developed and utilized.

## Materials and methods

### Cell culture

Three standard cell lines were used to investigate the performance of this method:

• MCF-7, a human breast cancer cell line (European Collection of Cell Culture).

• U-343, a human glioma cell line (Westermark et al 1973).

• BON, a human neuroendocrine tumor cell line (a kind gift from Dr. C.M Townsend, University of Texas, Galvestone, USA) derived from a lymph node metastasis of a pancreatic carcinoid.

The MCF-7 cells were grown in MEM-Eagle medium supplemented with 10% FCS, 1 mM sodium pyruvate, 2 mM L-glutamine, 1% non-essential amino acids and 5% Penicillin (Tamro). U-343 cells were cultured in Ham's F-10 medium supplemented with 10% FCS, 1 mM sodium pyruvate and 2 mM L-glutamine (Tamro). BON cells were grown in Ham F-12 K medium (NordCell, Sweden) mixed with DMEM medium supplemented with 10% FCS, 1 mM sodium pyruvate and 2 mM L-glutamine (Tamro).

The medium was changed twice a week and the cells were maintained in exponential growth phase.

### Multicellular tumor culture

The tumor cells were trypsinized from the stem monolayer culture, then cell suspensions were seeded in 24-well 1% agarose coated culture plates, with approximately 50,000 cells per well for U-343 and MCF-7 and 15,000 for BON. The cultures were kept at 37°C with 5% CO_2_, and grown for a period that depended on duplication rate for each cell line [6].

MTS medium for MCF-7 was DMEM supplemented with 10% FCS, 1 mM sodium pyruvate, 2 mM L-glutamine, 1% non-essential amino acids, 5% Penicillin (Tamro), 0.01 mg/ml Insulin and 1 nM β-Estradiol (Sigma Aldrich).

### Image Analysis

The aggregates were photographed daily using a Nikon Colorpix 4500  digital camera mounted on a Zeiss Axiovert 135 Microscope . The digital camera was set to use up to 4.0 million effective pixels, with an image resolution of 640 × 480, auto-focus, auto-shooting modes, with applied "Hi" image quality. A 5× magnification objective, 5x/0,12; 44 01 20 on Zeiss Axiovert 135 Microscope was used with adjustment of the strength of background light depending on the color of the medium that was used for growing the desired MTS.

Images were saved as sequences of JPEG files in a 128 MB, Scan Disc Compact Flash card and transferred into the hard disc of a personal computer with installed Windows 2000 for further image and statistical analysis.

**Figure 1:**
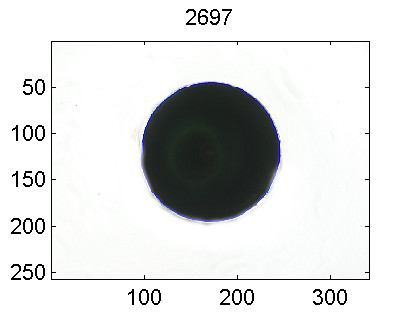
Reference object used for absolute calibration of area. The units on the axis are number of pixels.

A software program in Matlab (The Mathworks, Natick, Massachusetts) with user-friendly interface was developed to perform the image analysis using routines from the "image processing" toolbox. For absolute calibration of the area, a spherical object with a specified diameter of 0.79375 *μm*, as shown in Fig. 1, was used as reference object.

After feeding some input parameters such as name of starting image, number of images to analyze, project name, the software automatically delineated a ROI (Region of Interest) discriminating the necrosis area from the area where the cells were still alive and a ROI around the whole MCT. This was done automatically and sequentially for all images. The total area of the MTS and total area of necrosis were calculated as the total number of pixels inside the ROIs and was converted into *μm*^2 ^by using the radius of the reference object. To calculate the volume of the MTS the following equation was used.

V=43πr3
 MathType@MTEF@5@5@+=feaafiart1ev1aaatCvAUfKttLearuWrP9MDH5MBPbIqV92AaeXatLxBI9gBaebbnrfifHhDYfgasaacH8akY=wiFfYdH8Gipec8Eeeu0xXdbba9frFj0=OqFfea0dXdd9vqai=hGuQ8kuc9pgc9s8qqaq=dirpe0xb9q8qiLsFr0=vr0=vr0dc8meaabaqaciGacaGaaeqabaqabeGadaaakeaacqWGwbGvcqGH9aqpdaWcaaqaaiabisda0aqaaiabiodaZaaacqaHapaCcqWGYbGCdaahaaWcbeqaaiabiodaZaaaaaa@352E@

Where r=area/π
 MathType@MTEF@5@5@+=feaafiart1ev1aaatCvAUfKttLearuWrP9MDH5MBPbIqV92AaeXatLxBI9gBaebbnrfifHhDYfgasaacH8akY=wiFfYdH8Gipec8Eeeu0xXdbba9frFj0=OqFfea0dXdd9vqai=hGuQ8kuc9pgc9s8qqaq=dirpe0xb9q8qiLsFr0=vr0=vr0dc8meaabaqaciGacaGaaeqabaqabeGadaaakeaacqWGYbGCcqGH9aqpdaGcaaqaaiabdggaHjabdkhaYjabdwgaLjabdggaHjabc+caViabec8aWbWcbeaaaaa@3735@ was used to calculate the radius of the sphere. The total volume of the necrosis was calculated and subtracted from the total volume of the MTS to obtain the total volume of the living part. Moreover the fraction of total volume constituted by living cells was calculated.

All images with outlined ROIs discriminating the different parts of the MTSs were visualized and automatically saved as TIFF (Tagged Image File Format) for further observations. All desired statistical measurements including the total volume of the whole MTSs and total volume of the necrosis part were saved in an EXCEL file with specified file name for the full group of MTSs.

Validation of method was performed by analyzing pictures from different angles of the same aggregate. The growth curves for the three selected cell lines were generated to illustrate the use of the program. During the first few days of culture after seeding, the aggregates settle, which means they do not follow an exponential growth pattern. The growth curves after this period of 5–9 days were plotted and data were fitted to a monoexponential to indicate the volume doubling time.

### Frozen Section Autoradiography and Staining

At a selected time point some of the MTSs were incubated with ^18^F-labeled Fluoro-Deoxy-Glucose (FDG) to confirm the extent of the necrosis part. After 40 min incubation with 100 MBq/ml FDG and washing for 3*5 min, the MTSs were frozen quickly at -20°C and sectioned for autoradiography [7].

The slices with a thickness of 25 μm were exposed on a phosphor-imaging plate for 20 h. Scanning and imaging were performed using software Image Quant (Molecular Dynamics, Sunnyvale, CA).

For further verification Hematoxylin & Eosin staining of the slices was used. This complementary part was to clarify some histological features in the MTSs.

## Results

**Figure 2:**
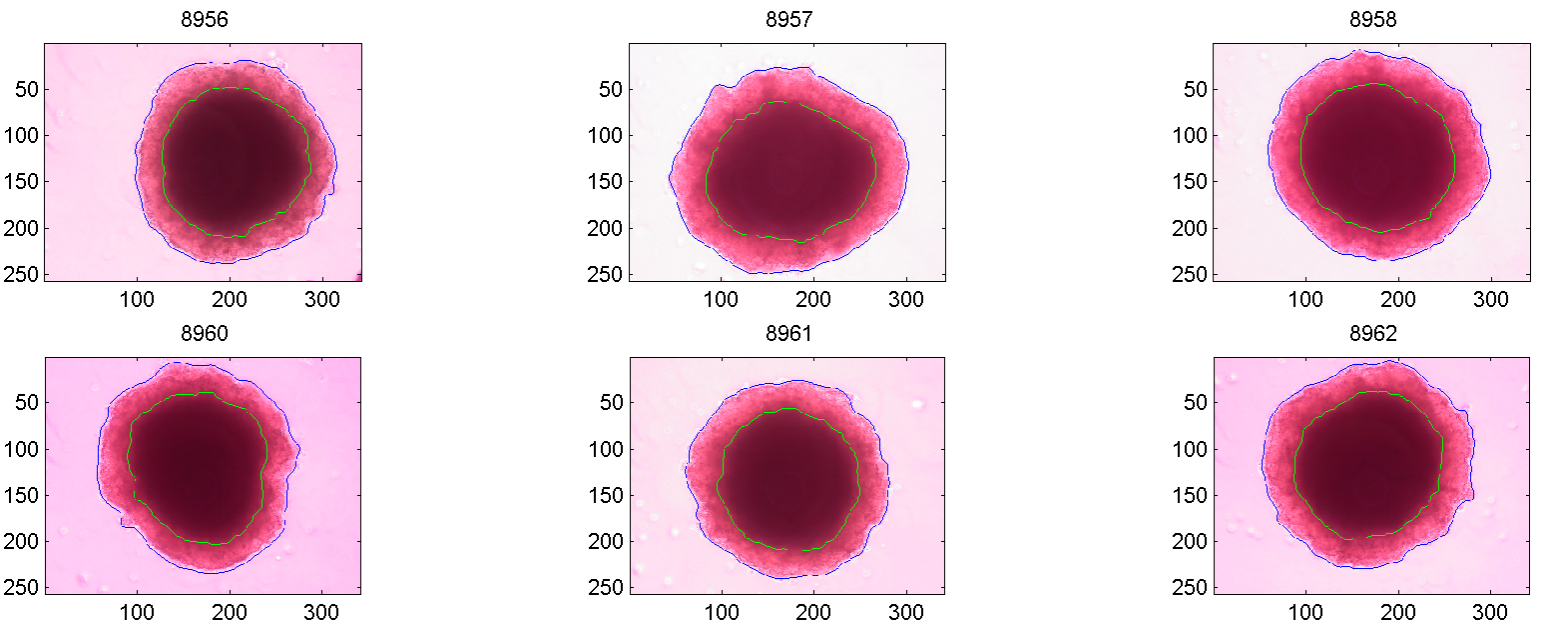
Images of six different MCF-7 cell aggregates. ROIs have been drawn separating the necrosis and living part from the background. The units on the axis are number of pixels.

**Figure 4:**
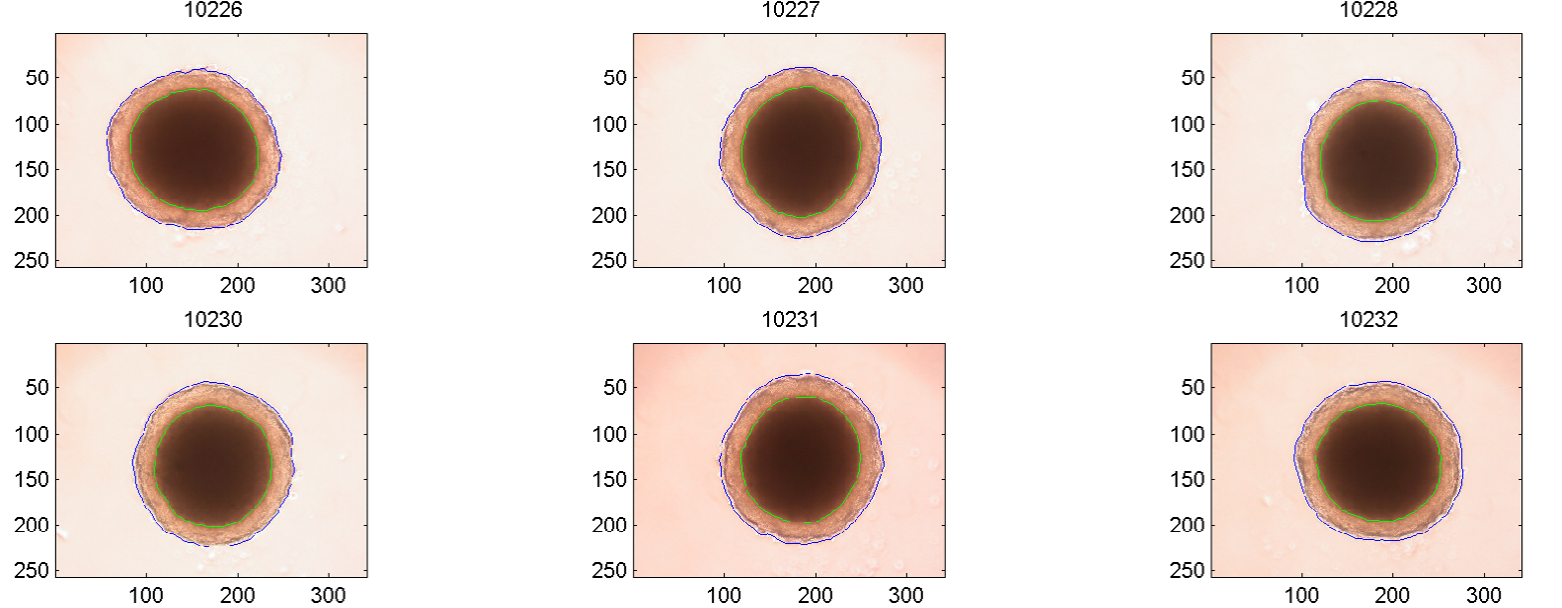
Images of six different U-343 MTS. ROIs have been drawn separating the necrosis and living part from the background.

**Figure 6:**
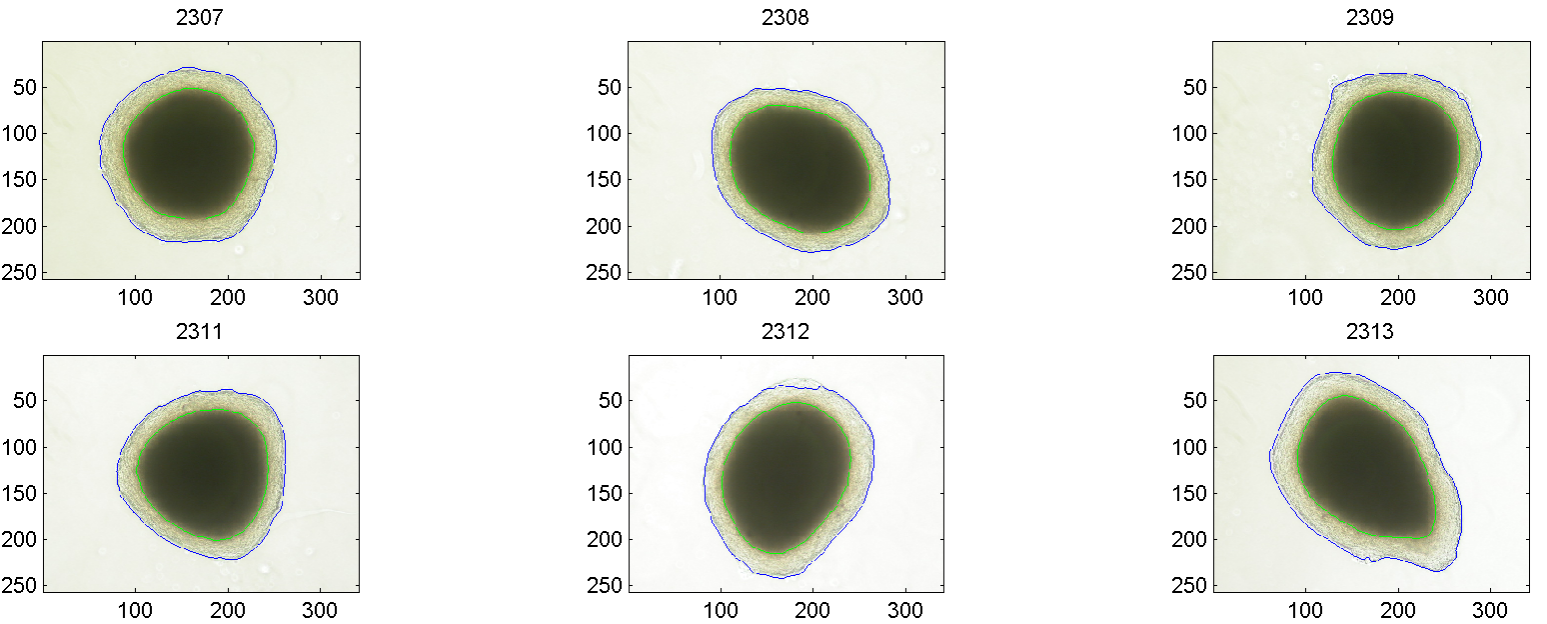
Images of BON cell aggregates. ROIs have been drawn separating the necrosis and living part from the background.

Multicellular aggregates from all cell lines directly after seeding showed a uniform feature without necrosis, which after a few days changed to the typical feature shown in Figure 2, 4, and 6, with a rim of viable cells surrounding a central necrosis. The shapes differed slightly as did the sizes of individual aggregates. The automatic outlining of the necrosis and the total periphery behaved properly in all cases.

The computation time for a set of 25 images was 17 s.

A reproducibility test of the method by repetitive measurements on the same aggregates, after shaking and rotation, showed a variability (Coefficient Of Variance) below 5%.

**Figure 3:**
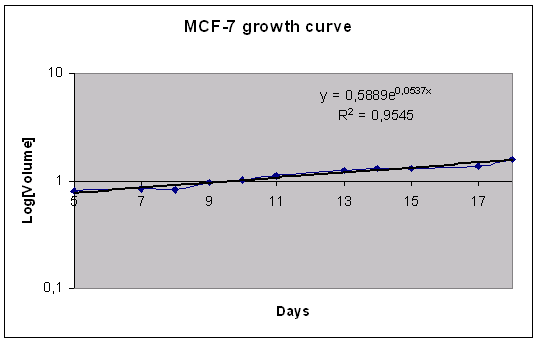
Volume growth pattern for the MTS of MCF-7 cell line during 17 days of observation. The data represent the mean of 24 MTS.

**Figure 5:**
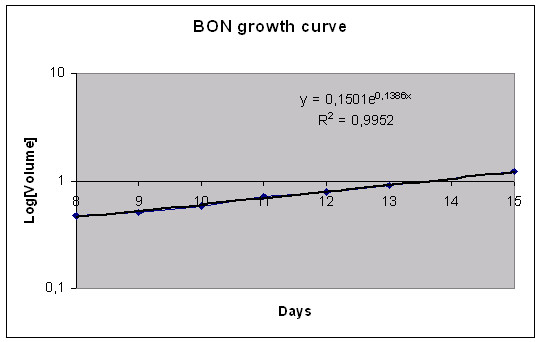
olume growth pattern for the MTS of U-343 cell line during 15 days of observation. The data represent the mean of 24 MTS.

**Figure 7:**
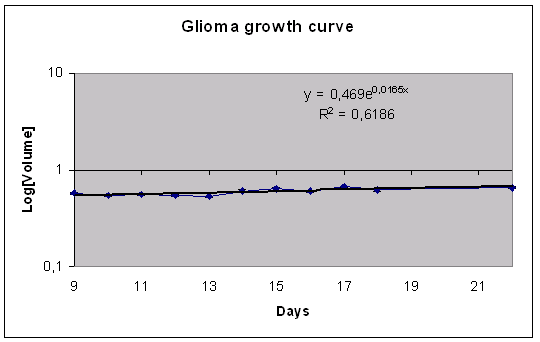
Volume growth pattern for the MTS of BON cell line during 17 days of observation. The data represent the mean of 24 MTS.

The MTS volume doubling time was calculated to be 13 days for MCF-7 (Figure 3), 57 days for U-343 (Figure 5) and 5 days for BON (Figure 7).

For each cell line, the Coefficient Of Variance within the group was below 15% but considerably higher in-between groups. This further confirms how essential it is to determinate the size of MTS for each experiment set-up.

**Figure 8:**
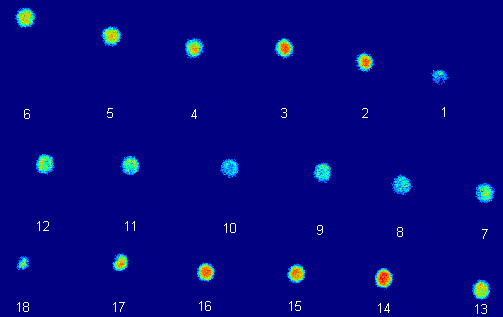
Frozen section autoradiography of an MCF-7 MTS incubated with ^18^F-FDG. Serial 25 μm sections are numbered from the top of the MTS to the bottom.

**Figure 9:**
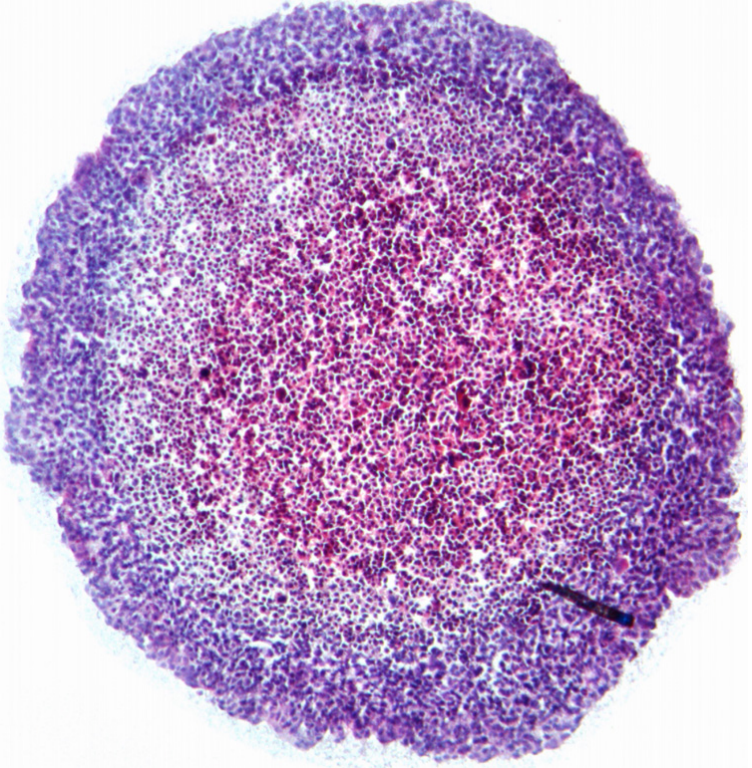
Hemotoxylin and Eosin staining of a BON MTS section. A focus of necrosis is seen in the middle part of the picture.

The result of autoradiography failed to show a clear rim of radioactivity, indicating that the resolution possible with a PET tracer is not sufficient for this task, whereas H&E staining confirmed the necrosis part in the middle of the MTSs (Figure 8, 9).

## Discussion

The traditional method to use cultured tumor cells for biology evaluations, including assessment of radiotracer uptake and effects of treatment, uses monolayer culture. This method is easy to apply, allows a good and reproducible source of cells and allows easy calculation of cell number by counting with sophisticated methods. However, there are some clear drawbacks to the method, especially for evaluating radiotracer uptake and monitoring of treatment response. The primary issue is that the growth is severely up-regulated in monolayer as compared to in vivo growth due to lack of adequate contact inhibition. This accelerated growth and lack of direct communication with neighboring cells also questions the physiological relevance, with the expectation that cellular function is aberrant. Furthermore, a monolayer with its rapid growth, quickly changes from a sparse cell culture to a culture with overlay growth, and thereby prohibits long-term follow-up of normal growth pattern compared to growth with pharmacological modulation. To overcome this, it is customary to only study short-term effects or to expose the cells to trypsinization and reseeding, a process that can induce further changes to cellular function.

For radiotracer uptake studies, we have since long been working with multicellular aggregates, grown on agar as a method to have easy access to cells. This method has the additional advantage that it allows easy washing of aggregates followed by a measurement of radiotracer uptake, after which the aggregates can be returned to culture for subsequent evaluations if the radiotracer is a PET tracer with short half-life.

A prerequisite to properly evaluate cell physiology with PET tracers is that the uptake value is related to the amount of living cells, especially in evaluations of treatment effects where changes in the relative proportion of viable cells can be expected. We have therefore developed a routine including quantization of PET tracer uptake that relies on accurately measuring viable cell volume.

The introduced method is effective, user-friendly, and time efficient, and is more accurate than the traditional method. It is known that a tumor cell-line can behave differently under different conditions. One of the advantages of this method is that a growth curve can be generated simply for each cell-line in each laboratory and for each experimental set-up.

Combining SASDM with biological evaluation of the MTS provides a good model to evaluate the effect of various cancer treatments e.g. chemotherapy and radiotherapy.

## Conclusion

To conclude, the combination of PET radiotracers and image analysis in MTSs provides a good model to evaluate the relationship between viable volume of tumor and the uptake of metabolic tracer before and after chemotherapy. This feature could be used for a selection of PET biomarker for an early assessment of treatment response.

## Competing interests

The author(s) declare that they have no competing interests.

## Authors' contributions

Authors AM, PR and MB helped with the design of the study. They created the method for applying SASDM, performed the image and data analysis and drafted the manuscript.

Authors RJ, ÖL and BL helped with some of the practical approaches and the writing of the paper.
